# Calcium imaging for analgesic drug discovery

**DOI:** 10.1016/j.ynpai.2021.100083

**Published:** 2022-01-05

**Authors:** Federico Iseppon, John E. Linley, John N. Wood

**Affiliations:** a Molecular Nociception Group, Wolfson Institute for Biomedical Research, University College London, Gower Street, WC1E 6BT London, UK; b Discovery UK, Neuroscience, Biopharmaceuticals R&D, AstraZeneca, Cambridge, UK

**Keywords:** Calcium imaging, *In Vivo* experiments, Pain research, Preclinical research, Analgesic drug discovery

## Abstract

Somatosensation and pain are complex phenomena involving a rangeofspecialised cell types forming different circuits within the peripheral and central nervous systems. In recent decades, advances in the investigation of these networks, as well as their function in sensation, resulted from the constant evolution of electrophysiology and imaging techniques to allow the observation of cellular activity at the population level both *in vitro* and *in vivo*. Genetically encoded indicators of neuronal activity, combined with recent advances in DNA engineering and modern microscopy, offer powerful tools to dissect and visualise the activity of specific neuronal subpopulations with high spatial and temporal resolution. In recent years various groups developed *in vivo* imaging techniques to image calcium transients in the dorsal root ganglia, the spinal cord and the brain of anesthetised and awake, behaving animals to address fundamental questions in both the physiology and pathophysiology of somatosensation and pain. This approach, besides giving unprecedented details on the circuitry of innocuous and painful sensation, can be a very powerful tool for pharmacological research, from the characterisation of new potential drugs to the discovery of new, druggable targets within specific neuronal subpopulations. Here we summarise recent developments in calcium imaging for pain research, discuss technical challenges and advances, and examine the potential positive impact of this technique in early preclinical phases of the analgesic drug discovery process.

## Highlights

• Calcium imaging is an efficient way to dissect the activity of neurons *in vivo*.
• GCaMP indicators can be expressed in specific cell populations for *in vivo* imaging.
• Pain research have benefitted greatly from these features in the recent decade.
• Preclinical research is shifting towards the analysis of pain models and mechanisms.
• *In vivo* calcium imaging is the ideal tool for an efficient drug discovery paradigm.

## Introduction

Within the mammalian nervous system, neuronal and glial cells are organised in defined networks and communicate at different spatial and temporal scales to convey sensory information from the outside world to the brain. Sensory perception, both innocuous and painful, is a very complex phenomenon. The different thermal, mechanical, and chemical stimuli need to be correctly transduced by the peripheral afferents and the information delivered centrally to the brain to elaborate an appropriate behavioural response (Todd, 2010). One of the major challenges of modern neuroscience is indeed the constant development of tools and techniques that allow the observation and the recording of signalling within this network at the relevant spatio-temporal scales and with environmental conditions that retain the physiological characteristics of an intact, behaving animal as much as possible.

The principal technique used to record and analyse the physiological responses of neurons and axons is electrophysiology. Intracellular and extracellular recordings are still widely used to assess the responses of single and multiple neuronal cells by recording the electric activity of single cells as well as local field potential generated by integrating the responses from multiple nearby neurons (Hooper and Schmidt, 2017). Field potentials, as well as single cell APs can be recorded simultaneously *in vivo* in selected brain regions as well as peripheral structures such as the dorsal root ganglia (DRGs) with multi-electrode arrays to isolate the activity of single and multiple neurons in response to electrical stimuli of peripheral nerves (Kashkoush et al., 2019), and these techniques may be used alongside optical approaches to further specify the contributions of different neuron populations (Chen et al., 2019b, Ma et al., 2010).

Another staple electrophysiology technique widely used *in vitro* and *ex vivo*, whole cell patch clamp, has been also used extensively *in vivo* in anesthetised and awake animals (Petersen, 2017). It usually implies blind approaches to the tissue of interest (central or peripheral) to record neuronal activity, but it can be coupled with two-photon imaging and optogenetics to have the possibility to select and influence the activity of specific neuronal sub-populations, albeit one or few cells at a time. This approach can be further potentiated by performing simultaneous dual cell recordings to assess correlations between responses of neurons belonging to the same or different population (Margrie et al., 2003, Petersen, 2017).

Another interesting, novel approach is the recording of Compound Action Potentials (CAPs) on peripheral nerves (Parker et al., 2018). CAPs are the summation of all the single fibres responding to a stimulus delivered on the nerve and a measure of the capability of such nerve to transmit sensory information centrally. These stimuli are recorded through cuff electrodes placed around the nerve and have a particularly interesting use in pain and sensory perception studies, since factors like chronic or neuropathic pain can rapidly alter the characteristics of nerve conduction as well as its velocity and can be rapidly detected by this method (Koh et al., 2019, Parker et al., 2018).

Electrophysiology techniques allow the measurement of properties of specific kinetics of action potentials, the isolation and studies of ion species and channels involved in the neural activity, specific cellular dynamics like synaptic plasticity with a precision still unmatched: they are still a staple for the study of neuronal activity both *in vitro* and *in vivo*. However, alongside the advantages, have their limitations: one of the most evident is the difficulty to record the activity of multiple neurons simultaneously. Where this can be achieved by using multi-electrode arrays or recording CAPs, there is a price to pay in terms of spatial resolution and the inability to precisely pinpoint the single cells being recorded. These drawbacks were therefore the drive for the development of imaging techniques where the electrical activity of large population of neurons can be monitored (directly or indirectly) *in vitro* and *in vivo* within anesthetised or awake animals with single-cell spatial resolution (Broussard et al., 2014, Knöpfel, 2012, Petersen, 2017).

Genetically encoded indicators have now been developed for a multitude of proteins to monitor essential biochemical functions of the neural cells, from neurotransmission to membrane potential, calcium and voltage dynamics, vesicle trafficking and receptor mobilisation among others (Broussard et al., 2014, Russell, 2011). The focus of this review are the advances in the imaging of calcium transients as an indirect measure of neuronal excitability with the help of Genetically Encoded Calcium Indicators (GECIs), with a spotlight on GCaMP probes. This technique has allowed the recording of large population of neurons simultaneously over different spatial and temporal scales *in vitro, ex vivo* and *in vivo* (Knöpfel, 2012, Russell, 2011)*.* This review is primarily focused on *in vivo* calcium imaging of peripheral structures involved in somatosensation and pain. Indeed, DRGs and other peripheral ganglia can be exposed for direct imaging of anesthetised and awake animals while an innocuous or noxious stimulus (mechanical, thermal, or chemical) can be applied to the peripheral structures innervating these ganglia, allowing for live recording and analysis of the activity of large population of neurons (Anderson et al., 2018). These tools, coupled with the development of multiple animal pain models and the more and more precise genetic profiling of defined neuronal subpopulations, are fundamental for the dissection of the complex circuitry involved in somatosensation and pain, as well as the molecules that play a paramount role in physiological and pathological signal transmission.

Moreover, pain is a complex condition, with many different aetiologies and time courses: this heterogeneity of clinical conditions has made it difficult to select single tractable targets with broad efficacy over multiple affections (Woolf, 2010). Widespread, chronic pain is widely diffused within the general population and difficult to treat, with currently available analgesic drugs that appear to be moderately effective at best while often producing unbearable side effects or long-term safety issues (Breivik et al., 2006, Dahlhamer et al., 2018). The pharmacological investigation and production of new, effective analgesic drugs is therefore an ever changing and difficult research field that takes advantage, especially during the early developing stages, on the achievements of basic pain research to understand the circuitry as well as to find molecular targets for therapeutic purposes (Woolf, 2010, Yekkirala et al., 2017). In this context, the possibility to image the activity of defined neuronal populations and any changes occurring upon pathological pain conditions as well as drug treatment is a powerful tool that has been and can be further used to investigate the function, dynamics, and site of action of newly developed analgesic drugs (Taneja et al., 2017). In this review, we provide evidence of the extensive use of this technique in somatosensation and pain basic research to dissect the role of definite molecules and cell populations that has led to the development of new therapeutic strategies and show promise for the application of novel approaches in early analgesic drug discovery and validation.

## Calcium imaging overview

The first attempt to image calcium transients dates back more than 50 years, when a calcium-sensitive bioluminescent protein was injected into cells to detect changes in intracellular calcium upon muscle fibre contraction (Ashley and Ridgway, 1968). Since then, progress has been made in the design of molecular calcium dyes, with the development of stable calcium dyes like Fura-2 and Oregon Green 488 BAPTA being just few examples of such dyes, whose wavelengths spans throughout the visible range (Takahashi et al., 1999). They can be easily loaded into cultured cells *in vitro*, a tissue slice *ex vivo* or a tissue *in vivo*, and simultaneously load many cells that can be imaged under a fluorescent microscope (Paredes et al., 2008).

These indicators, by definition, buffer calcium ions, and can thus alter physiological signalling: the affinity for calcium is therefore a fundamental characteristic of each indicator, as well as its velocity of calcium binding, that must be taken carefully into consideration when choosing which one to use in an experiment. These features are described by their constant of association (K_a_) and dissociation (K_d_), that are known, but most of the times must be empirically calculated, since they depend on the experimental environment (Paredes et al., 2008, Takahashi et al., 1999, Woodruff et al., 2002). Moreover, the spectral properties of these dyes must be object of careful consideration: they can be classified as ratiometric and single wavelength. The former presents a shift in either their emission or excitation peak wavelength upon calcium binding and release: this duality allows for an accurate measure of calcium concentration that is corrected for uneven dye loading, differences in cell volume, photobleaching, and dye loss (Lipp et al., 1996, Paredes et al., 2008, Schild et al., 1994). The latter cannot give such a precise quantification, but they minimize spectral overlap with other fluorophores and thus are easier to use. Moreover, the ratiometric dyes which change the excitation wavelength are more complex to use than the ones which change the emission peak and require a dedicated instrument. The loading in the cell is also another important aspect of these indicators. They come in different chemical forms: salts, dextran conjugates and acetoxymethyl (AM) esters. The first two forms are membrane impermeable and thus demand invasive loading procedures like electroporation or microinjection, limiting the amount of acquisition time and cells that can be imaged at the same time (Paredes et al., 2008, Takahashi et al., 1999). The AM esters were designed to correct these disadvantages and offer a quick and non-invasive loading into the cells, and long imaging time, since the intracellular esterases remove the AM group trapping the dyes inside the cells. These dyes, however, lack some fundamental characteristics to be successfully used for *in vivo* imaging. Their high background fluorescence, partially due to the lack of genetic control and cellular specificity, reduce the quality of the imaging, already weakened by the high light scattering of tissues, in addition to the fact that their highly invasive impact on the viability of the tissue imaged is not compatible with prolonged or chronic imaging (Broussard et al., 2014, Polikov et al., 2005, Stosiek et al., 2003).

GECIs based on fluorescent proteins like Green Fluorescent Protein (GFP) were developed to overcome these limitations: briefly, they are chimeric constructs of fluorescent proteins and calcium-binding proteins, engineered to be genetically targeted to specific cell populations or tissues using specific promoters (Whitaker, 2010). Some of these indicators were fused with two fluorescent proteins of different wavelengths (Chameleon probes) to employ the Förster Resonance Energy Transfer (FRET) phenomenon and thus a ratiometric, precise indication of subcellular calcium concentration (Miyawaki et al., 1997, Whitaker, 2010). The very first and most used class of GECIs is based on circular permutations of the GFP molecule, where mutations in the chromophore region yielded probes which reversibly changed fluorescence intensity levels based on changes in calcium concentration. Alongside PeriCAMs, the other type of permutated GFP indicators are the GCaMP sensors. This novel design resulted in a dramatic increase in fluorescence upon calcium binding with respect to previously available single wavelength and FRET-based probes, and their use in *in vitro*, *ex vivo* and particularly *in vivo* imaging rapidly increased (Nagai et al., 2001, Nakai et al., 2001, Whitaker, 2010). The constant striving for the improvement of the spectral characteristics of the GCaMP construct has led to the development of a large array of different probes with distinct kinetics, arriving in the latest years to the release of GCaMP-6, 7 and 8 variants (Chen et al., 2013, Dana et al., 2019; “jGCaMP8 Fast Genetically Encoded Calcium Indicators,” n.d.). Their high photostability, brightness and dynamic range to improve their signal-to-noise ratio by molecular engineering refinement have made GECIs, and GCaMP in particular, the most widely used probes for *in vivo* imaging in numerous model systems such as worm, zebrafish, flies and rodents (Dana et al., 2019, Guo et al., 2009, Higashijima et al., 2003, Tian et al., 2009). In the following sections, we will describe the biochemistry of the GCaMP probes, the technical instrumentation, and the challenges of *in vivo* calcium imaging, as well as the fundamental role that this technique played in the advance of the somatosensation and pain research fields.

## Biochemical properties and engineering of GCaMP indicators

**Fig. 1:**
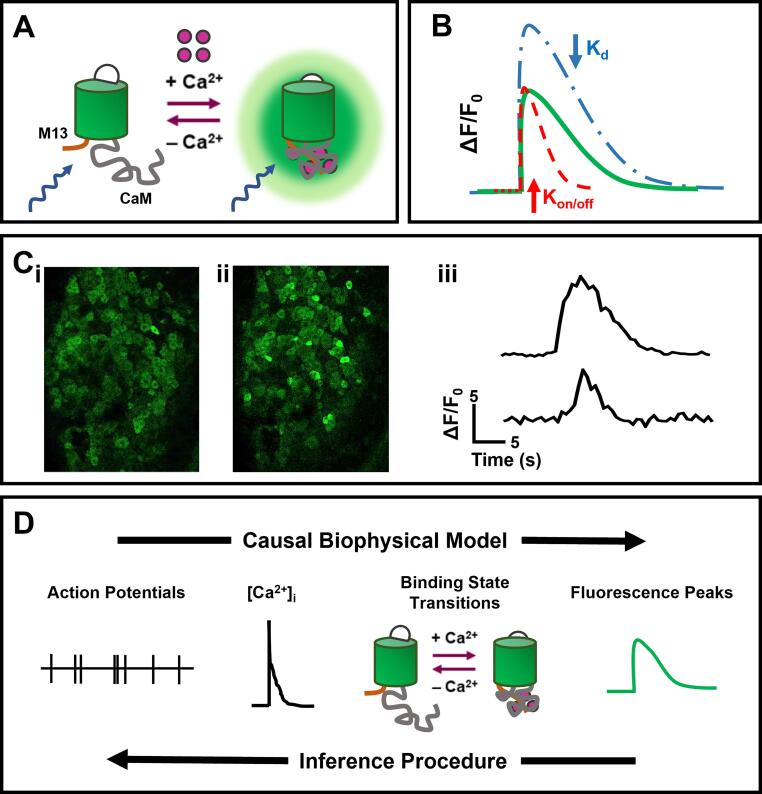
*GCaMP biochemistry.***A** Schematic representation of the biochemical structure of GCaMP probes and the conformational change occurring upon Ca^2+^ binding (A**i**). **B** Schematic representation of a GCaMP fluorescence trace and the changes that occur when using a sensor with higher affinity for calcium (lower K_d_, blue trace) and higher association/dissociation constants (K_on/off_, red trace). **C** Representative *in vivo* images of a DRG from a *Pirt-*GCaMP-3 mouse before (C**i**) and after (C**ii**) noxious heat stimulation. The traces in (C**iii**) are example traces from cells responding to noxious heat stimulation. **D** Conceptual illustration of a biophysical modelling used to link calcium fluorescence and action potential firing (adapted from Greenberg et al., 2018). The changes in calcium concentration occurring from neuronal activity are linked by a causal biophysical model. The binding transitions are modelled with highly complex mathematical algorithms and the parameters for individual GECIs and neurons are determined empirically. Finally, action potentials can be predicted from fluorescence data through and inference procedure. (For interpretation of the references to colour in this figure legend, the reader is referred to the web version of this article.)

GCaMPs are a family of GECIs in which the calcium binding domains are attached to one circularly permuted GFP molecule. Their structure is based on the calmodulin (CaM) calcium binding domain as the sensing element, which interacts with the calcium-CaM-binding motif M13 from the Myosin Light Chain Kinase protein (MCLK) and the circularly permuted GFP. The CaM motif can bind up to four calcium ions, triggering a conformational change that in turn enables binding to M13: these calcium-dependent interactions cause the de-protonation state of the GFP, altering the spectral properties of the chromophore to increase the intensity of GFP signal emission (Fig. 1A) (Akerboom et al., 2009, Anderson et al., 2018, Nakai et al., 2001).

The continuous endeavours in the design of these indicators resulted in the development of a plethora of different, improved versions of the original scaffold, which include GCaMP1.6, GCaMP2, GCaMP3, GCaMP-HS, Fast- GCaMPs, GCaMP5, and finally the GCaMP6, GCaMP7 and GCaMP8 series in the most recent years (Broussard et al., 2014, Chen et al., 2013, Dana et al., 2019; “jGCaMP8 Fast Genetically Encoded Calcium Indicators,” n.d.). Fundamental features which determine the efficacy and range of use of these GCaMP probes for *in vivo* experiments are sensitivity and kinetics, that in turn depend on biophysical characteristics of the probes. The sensitivity of an indicator is determined by its binding affinity for the calcium ions: higher affinity means higher sensitivity but slower response kinetics, since calcium ions stay attached to the probe for longer. The dissociation and association constants (κ_on_ and κ_off_) are essential for the rate of dissociation of calcium ions, and thus the kinetics of the probe itself, as it was the case for calcium dyes: faster kinetics mean lower decay time (the time taken by the indicator to return to resting fluorescence levels) that in turn result in higher temporal resolution of the probe itself (Fig. 1B). Faster probes (like GCaMP6f, GCaMP7f and GCaMP8f) have been able to resolve single APs in multiple tissues *in vivo*, albeit a trade-off between sensitivity and response times is often unavoidable and must be considered thoroughly for each experimental platform (Broussard et al., 2014, Chen et al., 2013, Emery et al., 2016; “jGCaMP8 Fast Genetically Encoded Calcium Indicators, xxxx, Tian et al., 2009). The first iterations of GCaMP probes had a very poor efficiency and performance compared to its parent protein GFP beyond 30 °C, making imaging in mammalian systems (at a physiological temperature of ∼ 37 °C) impossible (Whitaker, 2010). From GCaMP2 and 3 their stability, as well as their increased sensitivity, overcame the previous limitations becoming a very valuable tool for *in vivo* imaging (Fig. 1C) (Nagai et al., 2001, Nakai et al., 2001, Russell, 2011). However, these sensors still suffered from relatively low sensitivity and slow kinetics. Further mutagenesis studies of the interface between the CaM motif and the cpGFP fluorophore produced the GCaMP6 series of probes: GCaMP6s, 6 m and 6f, optimised for low, medium, and fast calcium signal kinetics respectively. GCaMP6s showed a more than tenfold improvement in sensitivity, whereas GCaMP6f was shown to be the fastest GECI for cytoplasmic free calcium in neurons (Chen et al., 2013). The continuous race to improve these sensors brought light to the latest probe series, GCaMP7 (GCaMP7s, 7f, 7b, 7c) and GCaMP8 (GCaMP8s, 8 m, 8f) with once again improved sensitivity and kinetics with respect to the GCaMP6 series, and with brightness and kinetic properties that were tailored to different, specific applications. The enhanced signal-to-noise ratio of these new probes will allow for *in vivo* imaging at higher speeds and over wider areas in the central and peripheral nervous systems than currently used probes such as GCaMP3 and 6, albeit having about the same dynamic range as the previous ones (Dana et al., 2019; “jGCaMP8 Fast Genetically Encoded Calcium Indicators,” n.d.). These probes can be used alone or in conjunction with animals expressing red fluorescent proteins like Tomato to highlight a specific neuronal subpopulation (Luiz et al., 2019). However, in the case of transgenic animals expressing GFP-based proteins, red fluorescent GECI variants, such as R-GECO based upon mApple and RCaMP based on mRuby, have been engineered to expand the possibilities of multichannel imaging (Dana et al., 2016, Zhao et al., 2011), enabling researchers to investigate the function of two distinct cell types simultaneously (Inoue et al., 2019, Jercog et al., 2016).

Although the potential of these probes is well-known, calcium signals remain an indirect measure of the electric activity of neurons. These probes are able to resolve the calcium spikes from single APs in neurons: applying single AP-like stimuli caused detectable changes in GCaMP-3 probe, and electrical stimulation of the hindpaws of mice that caused responses in the ranges of 1–3 APs were easily detected by the indicator *in vivo* in the DRGs, with a linear correlation between the number of APs and the GCaMP fluorescence intensity (Emery et al., 2016). However, their kinetics (even the ones of the fastest indicators), as well as the usual frequency of the image acquisition is in the range of 10–20 Hz. These frequencies cannot resolve single APs at the millisecond timescale that is needed and is possible to reach with electrophysiology. Moreover, this linear correlation between APs and calcium spikes is not present in all the neuron populations, and often the activities with multiple ranges of amplitudes and frequencies arising from the analysis of complex behaviours *in vivo* are challenging to resolve (Éltes et al., 2019, Wei et al., 2020). In the recent years there has been an effort for the inference of AP signals from GCaMP measures: this approach involves heavy pre- and post-processing of the raw acquired images, with the use of complex algorithms and neural networks to improve the signal-to-noise ratio (SNR), given that some biophysical characteristics of the indicators, like the peak amplitude and decay time, are known or quantifiable empirically (Fig. 1D) (Greenberg et al., 2018, Linsley et al., n.d.).

The optimal solution would be using direct indicators of transmembrane voltage changes: these voltage-sensitive probes are viewing a huge increase in popularity in the recent years, and more modern designs like ArcLight and ASAP1 show fast kinetics and sufficient brightness to detect sub-threshold potentials with milliseconds temporal resolution in hippocampal neurons *in vitro* (Broussard et al., 2014, St-Pierre et al., 2014). Unfortunately, the need for an imaging platform capable of acquiring images at more than 500 Hz, with the unavoidable shortcomings such as lower spatial resolution and restricted field of view, as well as the still low brightness and SNR of these probes strongly limit their *in vivo* applications, especially in tissues difficult to image like the peripheral ganglia and the spinal cord, albeit the field is everchanging and rapidly improving (Éltes et al., 2019, Lou et al., 2016). Thus, with respect to voltage imaging, calcium imaging remains the easiest way to reliably measure the activity of neurons with sufficient spatial and temporal resolution to analyse hundreds (and sometimes thousands) of cells *in vivo* over a large field of view in any central and peripheral tissue.

## Methodology

The development of GECIs as markers for neuronal activity represents one of the d more reliable ways to analyse the dynamics of neural cells and circuits *in vivo* without resorting to invasive techniques that perturb the tissue and cellular integrity. GCaMP probes changes in fluorescence indirectly indicate action potential firing of large population of neurons and allow for the *in vivo* detection of spatially distinct response profiles in the central and peripheral nervous systems, from brain to spinal cord to DRGs. This technique however poses numerous challenges from a technical point of view, and for an experiment to be successful meticulous consideration must be taken to correctly choose the microscope configuration, transduction methods, surgical procedures and offline post-processing and analysis suitable for the desired results.

### GCaMPs gene delivery approaches

The major advantage of GECIs over synthetic dyes is the possibility to target their expression in specific neuronal populations using specific promoters and targeting sequences, thus eliminating any background noise and perturbations coming from off-target fluorescence signals such as glial cells. This feature is particularly important in the study of somatosensation and pain in both the central and peripheral nervous system. In recent years the great development of single-cell RNA sequencing enabled the characterisation of multiple sub-population of neurons within the DRGs and the dorsal horn region of the spinal cord (Häring et al., 2018, Usoskin et al., 2015). These “functional atlases” can be exploited to mark individual sub-populations of interest using specific promoters or targeting sequences and dissect their functional role in pain and perception.

**Fig. 2:**
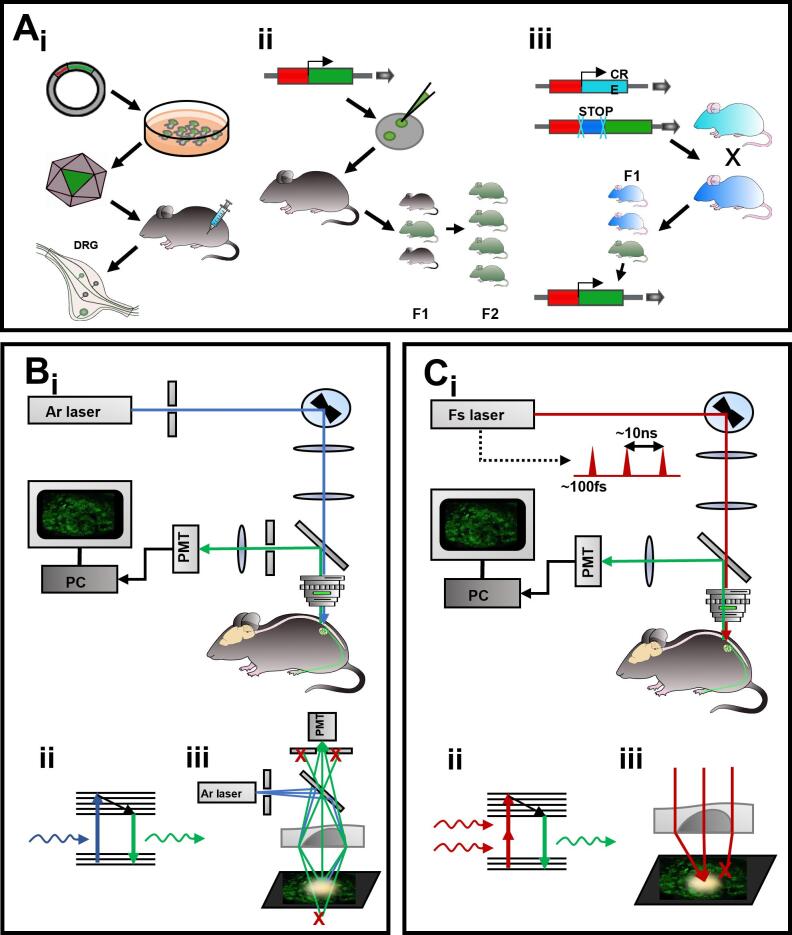
*GCaMP imaging: methodology and instrumentation.***A** Schematic representation of the different gene delivery methods commonly used for GCaMP probes: AAV injection (A**i**) in pups and adults is performed at different sites of the body (intraperitoneal, intrathecal, intraplantar); Transgenic mice (A**ii**) are produced with the injection of the probe into fertilized mouse oocytes or zygotes transplanted into a female carrier whose progeny will carry the gene of interest and can be then selected; The Cre/LoxP targeted recombination (A**iii**) offers high expression specificity and can be exploited either with mouse lines crossing or in combination with AAV specific injection. **B** Single photon confocal microscopy setup for *in vivo* imaging (B**i**). The light coming from the laser has lower wavelength and higher energy than the multi-photon approach, and it excites directly the fluorophore (B**ii**). The light that would reach the sample out of the focal plane is greatly reduced by two pinholes, whose diameter determines the depth of the focal plane itself (B**iii**). **C** Multi-photon microscopy setup for *in vivo* imaging of neuronal structures (C**i**). The short inter-pulse time ensures the time integration of multiple photons and the excitation of the fluorescent molecules (C**ii**) in the same focal plane and that greatly reduces out of focus excitation and allow the use of red-shifted, infrared wavelengths (C**iii**). (For interpretation of the references to colour in this figure legend, the reader is referred to the web version of this article.)

The two most common strategies for gene delivery of GECIs in specific cells involve the use of Adeno-Associated Viruses (AAVs) (Fig. 2Ai) or the creation of transgenic mice selectively expressing GECIs in neuronal sub-populations (Fig. 2Aii) (Jercog et al., 2016, Luiz et al., 2019, Walters et al., 2019). The AAV vectors are commonly used in optogenetic experiments, as well as for the delivery of a plethora of probes to monitor neuronal activity. Eleven different natural serotypes have been identified so far: each one differs from the other in its tropism, making them a very useful tool to preferentially infect a specific tissues (Burger et al., 2004, Haery et al., 2019, Wu et al., 2006). The transduction mechanism for AAV vectors is strongly dependent on the composition of the capsid proteins encapsulating the DNA, with small differences separating the various serotypes which differ in their optimal tropism towards different tissues and cell types both *in vitro* and *in vivo* (Castle et al., 2016, Haggerty et al., 2020). Short encoding sequences like GCaMP probes can be easily inserted in the vector with endogenous promoters that can target neuronal cells in a broad fashion, like synapsin-1 or thy1 proteins (Kuhn et al., 2012). AAVs 1,2, and 5 are broadly used to target neurons and glial cells at all levels of the brain and the spinal cord, with differences in transduction efficiency and cell types between the serotypes (Aschauer et al., 2013, Burger et al., 2004, Kuhn et al., 2012, Yu et al., 2016). Focusing on the sensory ganglia, the most common serotypes used are AAV1, 5 and 9, although other ones (6,7,8) are also able to express transgenes in sensory neurons: interestingly, AAV8 presents a particular tropism towards large-diameter DRG neurons, even without guidance promoters (Castle et al., 2016, Jacques et al., 2012). Genetic molecules can be transferred through intra-ganglionic direct injection, intrathecal injection into the cerebrospinal fluid, or via retrograde transfer injecting the viruses into the peripheral nerves and skeletal muscles, as well as intradermal into the hindpaws (for expression in the DRGs), whisker pads (for expression in the Trigeminal Ganglia) or intraperitoneal route (for general AAV expression) (Burger et al., 2004, Dang et al., 2017, Kuhn et al., 2012, Yu et al., 2016). The time course of expression varies based on the delivery route used, but generally spans from 4 to 12 weeks: it takes much longer time than the loading of chemical indicators but ensures a much more precise and long-lasting signal. The main technical advantage of targeting specific CNS and PNS areas with different serotypes is the plasticity that is offered to the experimenters, both in terms of efficiency and signal specificity in targeting specific cell populations in specific regions of the nervous system and in terms of choosing a delivery method that is optimal not only for the viral vector but for their experimental needs.

This method, however, has the disadvantage of a partial lack of specificity, due to the different expression within different tissues of the endogenous promoters utilised, as well as potential overlaps in expression of the same promoter by distinct neuronal subpopulations. One way to ensure specificity is to couple AAV injection and Cre/Lox-P mediated recombination (Fig. 2Aiii): transgenes inserted in recombinase-dependent viral vectors are injected, via the same routes used for normal AAV injections in transgenic animal lines where the Cre expression is restricted to specific neuronal subpopulations (Schnütgen et al., 2003). This strategy has been massively used to dissect the role of specific subsets of sensory neurons in the transmission of different innocuous and painful signals in the DRG (Luiz et al., 2019, MacDonald et al., 2021a, Walters et al., 2019). Transgenic targeting has been achieved in animals ranging from Drosophila to rodents and large mammals, including primates (Guo et al., 2009, O’Shea et al., 2017, Pologruto et al., 2004, Wang et al., 2003). Numerous transgenic rodent lines have been engineered to express various GECIs, like GCaMP2, GcaMP3 and GcaMP6s under control of different promoters like Thy1 or Pirt (Díez-García et al., 2005, Emery et al., 2016, Zariwala et al., 2012). Taken together, these strategies offer a wide choice to the researcher that has the possibility to select the approach that better suits his experimental needs in terms of expression efficiency as well as tissue and cell selectivity.

### Optical instrumentation

The optical system represents one of the predominant components in a successful *in vivo* fluorescence imaging experiment, particularly when imaging neural activity within specific regions of the nervous system. Each major region of interest (brain, spinal cord, DRG) retains different characteristics and requires diverse answers to the issues they pose in terms of spatial and temporal resolution, depth of imaging, light intensity, and image stability. The earliest recordings of *in vivo* calcium signals were done on invertebrates like *C. elegans* and small vertebrates like zebrafish embryos (Friedrich and Korsching, 1997, Jain et al., 1993, Ritter et al., 2001). These experiments used synthetic dyes and simple widefield microscopes using charge-coupled device (CCD) cameras exploiting the transparency of the tissues imaged. Today, due to the formidable advances in microscopic equipment, different avenues can be explored by researchers, each possessing its own advantages and disadvantages.

The two predominant imaging methods are one-photon (Fig. 2Bi) – either confocal or widefield – and multi-photon microscopy (Fig. 2Ci). The former employs the use of a high-energy light source to deliver excitation photons with wavelengths within the visible spectrum to the specimen (Fig. 2Bii, iii). The scattering of the short wavelength light strongly limits the depth of the tissue that can be imaged with single-cell spatial resolution to less than 100 μm, even though the use of CCD and complementary metal oxide semiconductor (CMOS) detectors can dramatically increase the temporal resolution to achieve kHz image acquisition rates (Helmchen and Denk, 2005). However, an important advantage of these platforms is that they are relatively easy to build and allow the imaging of spatially distinct response profiles from large sets of neurons challenged with numerous tactile and noxious mechanical and thermal stimuli *in vivo* in the superficial layers of the DRGs (Fig. 1C) (Emery et al., 2016, Yarmolinsky et al., 2016).

Multi-photon microscopy offers two fundamental advantages over single-photon illumination: the increase in the depth of imaging and the decrease in out of focus emission and light scattering. In this technique the photon absorption varies non-linearly with the density of photon excitation: this feature ensures that only the fluorophores in the focal plane of the excitation light are activated, and since multiple photons must combine their energy to excite a single fluorophore, longer wavelength, infrared light is commonly used (Fig. 2Cii, iii) (Denk et al., 1990). This ensures much lower phototoxicity and greatly increases the tissue depth routinely reachable with excellent spatial resolution to about 500 μm and in some cases over 1000 μm with three-photon microscopy (Helmchen and Denk, 2005, Nimmerjahn et al., 2008, Ouzounov et al., 2017). Nevertheless, traditional two-photon systems, widely used in *in vivo* imaging, use galvanometer mirrors, which greatly limit the acquisition speed and thus the frame rate. Furthermore, since the focal plane of acquisition is very constricted in the z-plane, the acquisition is very sensitive to motion artifacts, and therefore stabilisation of the tissue to be imaged is fundamental.

When planning an *in vivo* experiment, the choice of microscopy platform depends on the tissue to be imaged. In pain and sensation research, the tissues to investigate are mainly three: the brain, the dorsal horn of the spinal cord and the peripheral ganglia, ranging from DRGs to trigeminal ganglia. Imaging of specific cortical and sub-cortical regions within the brain require multi-photon imaging to reach the necessary depth within the tissue (Wang et al., 2018b); Equally, highly myelinated tissues like the spinal cord require two-photon microscopy, and positioning the animal under the microscope slightly rotated allows the exposure of more lateral portions of the dorsal horn and avoids the highly obstructive myelinated fibres which decrease resolution and signal-to-noise (SNR) ratio of the images (Johannssen and Helmchen, 2010, Xu and Dong, 2019). For smaller structures, like the peripheral ganglia, two-photon microscopy helps to image neurons deep within the tissue (Wang et al., 2018a); however, single photon confocal and widefield microscopes are sufficient to acquire images of the superficial cellular layers of whole ganglia with single cell resolution (Emery et al., 2016, Yarmolinsky et al., 2016).

Two key points to further consider when preparing an imaging experiment *in vivo* are the technical apparatus to restrain and hold the animal to ensure stabilisation during imaging and stimulation and whether the animal needs to be anesthetised or awake and behaving. Fixation of the animal to the stage can be done either on the head when imaging the brain and trigeminal ganglia or on the back for spinal cord and DRGs. This procedure locks the imaged tissue in position, almost eliminating the movements due to the heartbeat and breathing that cause motion artifacts and limit the spatial resolution. These corrections are vital especially in two-photon imaging, and furthermore when imaging peripheral regions like the spinal cord or the DRGs that are closer to the source of the movements. In acute experiments, movements can be compensated by fixing the column with firm metal clamps that elevate the animal enough to achieve stability (Sekiguchi et al., 2016, Xu and Dong, 2019). On the other hand, the surgical installation of a chamber implant fixed on the vertebrae, or a cranial window allow for chronic imaging of the same region of interest in the brain, spinal cord or DRG over multiple sessions (Broussard et al., 2014, Chen et al., 2019a, Tang et al., 2015; ).

Most of the *in vivo* calcium imaging experiments, above all in the pain research field, are performed under deep anaesthesia, to avoid motion artifacts due to unexpected movements and motor reflexes when the animals are challenged with noxious stimuli, while maintaining the physiological response to the sensory stimuli applied. The currently most used anaesthetics are isoflurane vapours, and the injection of urethane or cocktails of either ketamine + xylazine + acepromazine or ketamine + medetomidine (Emery et al., 2016, Johannssen and Helmchen, 2010, MacDonald et al., 2021a, Sekiguchi et al., 2016, Tang et al., 2015). However, certain anaesthetics like isoflurane have a physiological impact on the tissue, because of their mechanisms to achieve anaesthesia, often through suppression of excitatory transmission and subsequently calcium signals in neurons and glial cells ((Sekiguchi et al., 2016)). Therefore, it is necessary to strive to achieve imaging in awake, behaving animals. *In vivo* imaging of brain structures in awake animals has been achieved using head mounts to fix the animals or more recently with miniaturised microscopes that allow free movements of the animals and thus a concurrent assessment of behaviour and neural activity (Corder et al., 2019). The same approaches have been used to image neuronal and astrocyte activity in the sensory cortex of restricted and freely behaving mice (Kim et al., 2016a). Recently, the implementation of an implanted vertebral window allowed for long-term imaging of DRG neurons in awake behaving mice, without compromising the animals’ motor and sensory functions (Chen et al., 2019a).

As advances in microscopy technology and probe engineering proceed hand in hand, the possibilities for *in vivo* imaging of neural activity increases drastically. The miniaturised microscopes allow for imaging of awake, behaving animals, the incredible progresses in the optogenetic field boost the potential for simultaneous stimulation and recording of optical signals within central and peripheral structures of the nervous system, and the breakthroughs in micro- and nano-engineering lead the way to expand and integrate multiple features in emerging neural interfaces to convey electrical, optical and chemical signals to and from neural tissues (Frank et al., 2019).

## *In vivo* GCaMP imaging in pain research

The development of genetically encoded indicators of neuronal activity has greatly expanded the possibilities for neuroscience research in all fields, from functional organisation to short- and long-term plasticity of neural circuitry, especially in the brain. Pain, as already stated, is a complex phenomenon, with multiple anatomical and functional steps and different circuits embedded within all structures of the central and peripheral nervous system. The possibility to observe the response of these multiple neural circuits to innocuous and noxious stimuli *in vivo*, as well as the possibility to dissect the role of single neuronal cells and populations within these circuits, has greatly boosted the pain research field (Table 1). Of course, these structures are hard-to-reach targets, especially from an imaging point of view; however, the importance of studying these responses in an intact animal, ensuring the most physiological context possible, has urged researchers to overcome these issues.

**Table 1:** *GCaMP in vivo studies overview.* Different *in vivo* GCaMP experimental paradigms, with different delivery methods, tissues imaged, stimuli, and pain models used, as well as neuronal subpopulations studied.

**GCaMP Probe**	**Delivery Method**	**Imaged tissue**	**Stimulus**	**Pain model**	**Neuronal Subpopulations Investigated**	**Genetic Modification**	**References**
GCaMP3, GCaMP6s	Transgenic Mice (Pirt-GCaMP3, Rosa26-GCaMP6s)	DRG	Electrical, Mechanical, Thermal (Cold/Heat)	Inflammatory (PGE2)	None	None	Emery et al. 2016
GCaMP6s	AAV Injection (Intraplantar, Intrathecal)	DRG	Electrical, Mechanical, Thermal (Cold/Heat)	None	None	TRPV1 KI	Wang et al. 2018a
GCaMP6s	AAV Injection (Intrathecal)	DRG	Chemical (Formalin), Electrical, Mechanical, Thermal (Cold/Heat)	Inflammatory (UVB Irradiation)	None	None	Chisholm et al. 2018
GCaMP6s	Transgenic Mice (Thy-1-GCaMP6s)	DRG	None (Spontaneous Activity recording)	Inflammatory (Formalin)	None	None	Chen et al. 2019a
GCaMP3, GCaMP6s	Transgenic Mice (Pirt-GCaMP3, Pirt-GCaMP6s)	DRG	Mechanical	Inflammatory (CFA), Neuropathic (SN-CCI)	None	Cx43 KO	Kim et al. 2016b
GCaMP3	Transgenic Mice (Pirt-GCaMP3)	DRG	Mechanical, Thermal (Cold/Heat)	Neuropathic (Oxaliplatin, Ciguatera, PSL)	NaV1.8, CGRP, TrkB	NaV1.8 DTA (Ablation)	MacDonald et al. 2021a
GCaMP3	Transgenic Mice (E2a-GCaMP3)	DRG	Mechanical	Inflammatory (Inflammatory Mediators Cocktail)	None	None	Smith-Edwards et al., 2016
GCaMP6s	AAV Injection (Intrathecal)	DRG	Mechanical	Cancer (CIBP)	None	None	Kucharczyk et al. 2020
GCaMP3	Transgenic Mice (Pirt-GCaMP3/NaV1.8 Tomato)	DRG	Thermal (Cold/Heat)	None	NaV1.8	None	Luiz et al. 2019
GCaMP3, GCaMP6s	Transgenic Mice (Pirt-GCaMP3), AAV Injection (Intraplantar)	DRG	Mechanical, Thermal (Cold/Heat)	Inflammatory (PGE2)	None	NaV1.7 KO	MacDonald et al. 2021b
GCaMP6s	Transgenic Mice (Pirt-GCaMP6s)	DRG	None (Spontaneous Activity recording)	Neuropathic (SNL)	None	None	Chen et al. 2019b
GCaMP5A	Transgenic Mice (Rosa26-GCaMP5A, TRPV1-GCaMP5A, TRPA1-GCaMP5A, TRPM8-GCaMP5A)	TG	Thermal (Cold/Heat)	Inflammatory (Burn Injury)	TRPV1, TRPA1, TRPM8	TRPV1 KO	Yarmolinsky et al. 2016
GCaMP6s	Transgenic Mice (Thy-1-GCaMP6s)	Spinal Cord	None (Chronic calcium influx recording)	Spinal Cord Injury	None	None	Tang et al. 2015
GCaMP6s	AAV Injection (In-situ (Spinal Cord))	Spinal Cord	Mechanical	None	None	None	Sekiguchi et al. 2016
GCaMP6s	AAV Injection (In-situ (Dorsal Horn))	Spinal Cord	Mechanical	Neuropathic (SNI)	None	None	Chen et al. 2018
GCaMP56s	AAV Injection (In-situ (MCC))	Cortex (MCC)	Chemical (Formalin)	None	None	None	Hu et al. 2019
GCaMP6m	AAV Injection (In-situ (BLA))	Cortex (BLA)	Mechanical, Thermal (Cold/Heat)	Neuropathic (Sciatic NI)	None	None	Corder et al. 2019

The sensation of pain starts from peripheral nerve terminals that transduce the stimuli and transmit the information to the substantia gelatinosa (Laminae I and II) of the dorsal horn of the spinal cord (Peirs and Seal, 2016). The cell bodies of these peripheral neurons are located in the DRGs. In recent years, numerous groups, including ours, have investigated the dynamics of sensory and pain responses within the DRG of rodents and rodent pain models. Using these techniques, researchers have been able to monitor hundreds to thousands of neurons simultaneously in live, anesthetised, transgenic mice, where probes like GCaMP-3 and GcaMP-6 have been expressed within neuronal cells (Chisholm et al., 2018, Emery et al., 2016, Wang et al., 2018a). Recently, using a spinal window, a group managed to image for several weeks both the spontaneous and evoked activity dynamics of DRG neurons in awake, behaving mice (Chen et al., 2019a). This technique has been used to investigate the response profiles of large populations of neurons exposed to different mechanical and thermal stimuli and has revived the discussion about the polymodality of DRG neurons.

One study challenged the common view that most DRG neurons are intrinsically polymodal in their response to various stimuli: the data show that the great majority of DRG neurons are modality-specific, and polymodal response profiles are found in less than 15% of the neurons analysed (Emery et al., 2016). A different study reported a higher percentage of polymodal mechano-thermal sensitive neurons, as well as investigating the activity dynamics of these neurons under inflammatory conditions through the injection of formalin (Chisholm et al., 2018). Another study unravelled an unprecedented complexity in the thermal coding of DRG cells: they identified different strategies by which cutaneous temperature is represented in the DRG, with different response profiles found in different subsets of thermoceptive afferents (Wang et al., 2018a). A separate study found specific subpopulations of thermoceptive neurons in the trigeminal ganglion, where Transient Receptor Potential Vanilloid 1 (TRPV1) neurons are the main innocuous warm detectors, and at least three functionally diverse, Transient Receptor Potential Melastatin 8 (TRPM8) expressing neurons with different temperature thresholds which modulate cold perception (Yarmolinsky et al., 2016). This plethora of finely tuned modality specific and non-specific subpopulations of neurons dramatically enhance the complexity of the perception and coding of sensory and painful stimuli at the peripheral level.

These response profiles can be altered by inflammation and injury: PGE_2_ administration rapidly changes the response profile of these neurons and unmasks a large population of previously silent nociceptors (Emery et al., 2016). In addition, after injury, gap junction-mediated neuron coupling occurs in the DRG: this phenomenon occurs between cells of different diameter and innocuous stimuli may activate small-diameter nociceptors coupled to large diameter mechanically sensitive primary afferents, contributing to the occurrence of mechanical allodynia and chronic pain (Kim et al., 2016b). Injury indeed can transform the cellular representation and coding logic of these different populations in response to stimuli: following injury, TRPV1-expressing neurons are re-tuned with a shift of the temperature that represents noxious heat stimuli to normally warm temperatures. On the other hand, cold hypersensitivity seems to be driven by the recruiting new class of cold-sensing neurons (Yarmolinsky et al., 2016). Indeed, activation of normally silent, large-diameter cold sensing neurons is reported to be a common mechanism for cold allodynia in at least three clinically relevant, but etiologically different, neuropathic pain states (namely oxaliplatin-dependent peripheral neuropathy, ciguatoxin-2-dependent neuropathic pain, and partial sciatic ligation neuropathic pain models), derived possibly by a pathological change in the expression profile of specific ion channels (MacDonald et al., 2021a). Moreover, TRPV1-expressing neurons activated via heat stimuli greatly increases the number of silent afferents recruited during inflammation: these mechanically unresponsive neurons express TRPV1, and their activation following heat stimulus triggers the release of neuropeptides that combine with inflammation to trigger robust mechanical allodynia (Smith-Edwards et al., 2016). Similar changes in coding strategies and cell recruitment seem to occur also in cancer pain: rodent models of bone cancer pain develop robust mechanical hypersensitivity, that translates in an increased number of responsive cells through recruitment of previously silent, small nociceptive afferents, as well as a modification in the coding of pressure stimuli (Kucharczyk et al., 2020).

These discoveries depict a complex picture of distinct neuronal subpopulations and coding strategies underlying somatosensation and pain in both physiological and pathological environments. Indeed, a recent article used single-cell RNA sequencing, identified 11 distinct neuronal sub-populations within the DRGs, each presumably with a specific function in the coding and transmission of sensory information ( Usoskin et al., 2015, Wood et al., 2020b). Each cluster of cells can be theoretically isolated and targeted via transgenic modification, via the Cre/Lox-P method to investigate its role in sensory function. For example, various sodium channels isoforms are fundamental for action potential initiation and transmission and have been identified as major contributors to pain sensation (NaV1.7, Nav1.8, NaV1.9). Their silencing is related to various degrees of pain insensitivity, particularly in the case of Nav1.7 (Cox et al., 2006, Sexton et al., 2018, Wood and Iseppon, 2018). Using *in vivo* GCaMP imaging, in combination with selective deletion using transgenic KO mice, the role of NaV1.8-positive neurons in cold sensation has been explored and it was found that they are required for prolonged extreme cold sensing. Moreover, ablation of the NaV1.8-positive subpopulation increased the acute cold sensitivity of the mice to temperature higher than 5 °C, suggesting a possible disruption in temperature sensing and increase in aversive responses from the surviving cold-sensing neuronal population (Luiz et al., 2019). Deletion of the NaV1.7 gene SCN9A causes congenital pain insensitivity, and transgenic mice where NaV1.7 is knocked out through and Advillin-Cre promoter are pain-free in numerous mechanical and thermal assays (MacDonald et al., 2021b). However, calcium imaging *in vivo* in these mice showed that nociceptor excitability at the level of the DRG was largely unchanged, with only a 30% decrease in mechano-responsive neurons. The disruption seems to reside in the spinal cord, where the NaV1.7 KO DRG neurons have significantly increased synaptic threshold, and therefore seems fail to correctly transmit nociceptive information to central terminals in the dorsal horn (MacDonald et al., 2021b).

The advent of two-photon imaging allowed researchers to visualise fluorescent cells deeply embedded within the neural tissues: in the recent years this approach allowed the visualisation of activity of neurons and glia in the spinal cord (Xu and Dong, 2019). Albeit this approach still appears demanding, and some groups prefer to use organic dyes like Oregon Green 488 to image the spinal cord neurons (Ran et al., 2016), GCaMP probes can be used to image neurons and axons in the spinal cord of anesthetised and awake mice both acutely and repetitively with a chronic implantation of a spinal window ((Sekiguchi et al., 2016, Tang et al., 2015)). Furthermore, a recent study that combined two-photon imaging of the spinal cord with electrical stimulation of the anterior cingulate cortex (ACC) region of the brain has shown how this facilitatory descending circuit potentiates the response of mechano-sensitive neurons in the dorsal horn, and how the activation of this circuit produced pain sensitisation (Chen et al., 2018). The central circuits for pain cannot only be electrically stimulated, but also imaged to unravel the function of different microcircuits within the brain. Recently, using a combination of optogenetics and calcium imaging in awake, behaving mice, one group dissected the role of two distinct, functionally opposite neuronal subpopulations within the medial cingulate cortex (MCC), that facilitate and inhibit nociception in physiological conditions. Upon injury, there is a shift in activation pattern with a significant decrease of activity in the inhibitory population and the onset of mechanical hypersensitivity (Hu et al., 2019). Moreover, through the installation of a miniaturised microscope (Miniscope), another study demonstrated the paramount role of nociceptive neuronal ensembles within the basolateral amygdala (BLA) for the codification of the affective dimension of pain stimuli, without altering their sensory component (Corder et al., 2019).

Taken all together, these further results clearly highlight the enormous potential of this technique in unveiling the role of specific cells and circuits in innocuous and pain sensation at all levels in the nervous system, as well as the potential for discovery of new targets for the development of analgesic drugs and new therapies.

## Potential impact of calcium imaging in preclinical research

The sensation of pain is a unique phenomenon involving many different circuits and neuronal populations within the central and peripheral nervous system. Pain is not a unitary phenomenon and can be divided into four different major subtypes: nociceptive, inflammatory, neuropathic, and dysfunctional (Woolf, 2020). Pain can be the acute sensation that arises from the stimulation of the primary afferents scattered throughout the body and acts as a protective mechanism from putative causes of harm. However, maladaptive modifications of the circuitry due to external or internal causes, like inflammation, nerve injury, cancer (and cancer treatment), prolonged trauma or abnormal functionality of the nociceptive system, can evolve into chronic pain. This debilitating condition can present itself as an increased response to painful stimuli (hyperalgesia), a hypersensitivity to innocuous stimuli (allodynia), or a continuous, ongoing pain that persists even after the injury or treatment that caused it has long healed (Woolf, 2020, Yekkirala et al., 2017). Given the complexity of this phenomenon, its correct treatment becomes equally difficult: for many years the medical profession classified pain mainly based on its severity (mild, moderate, severe) and used these categories to select and utilise few classes of analgesics, mainly nonsteroidal analgesics, and opioids. However, the different molecular and cellular components, as well as the different circuits involved in pain physiology and pathology that have been discovered in the recent decades paint a totally different and multifaceted story, an issue that cannot be tackled with one or two “bestseller” analgesic drugs. These past mistakes in pain management contributed to an increase in opioid overuse, a crisis that hit especially hard in the United States (Singh et al., 2019, Skolnick, 2018). Therefore, the search for new, functional molecules that develop stable analgesia without liability to abuse and which can integrate with, or substitute opioids is urgent and paramount. Such advances in pain therapeutics are strongly dependent on the general understanding of the diverse mechanisms associated with specific pain conditions (King and Porreca, 2014, Woolf, 2010, Yekkirala et al., 2017).

The current drug discovery paradigm, comprised of a preclinical phase where multiple molecular candidates of specific targets are screened and positively selected to progress into a clinical phase of testing in human patients, has been extensively used by pharmaceutical companies. However, the last 10 years have exposed its multiple flaws in finding analgesics with novel mechanisms of action. The numerous failures, particularly when they occur in the latest phases of clinical trials, cost an enormous amount of money and time, and expose the urgent need to take novel approaches to target identification and validation in preclinical phase (Taneja et al., 2017, Woolf, 2020, Woolf, 2010).

**Fig. 3:**
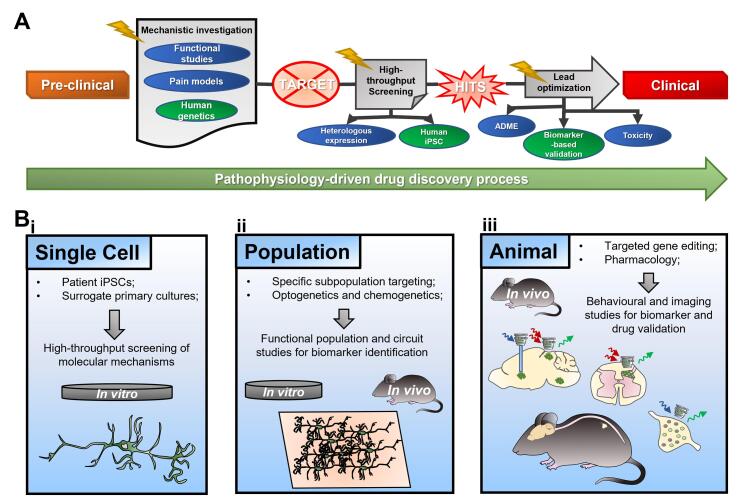
*Preclinical drug discovery process and the potential impact of GCaMP in its innovation*. **A** Schematization of the current preclinical drug discovery process. The target is identified via functional studies on surrogate pain models. Then a high throughput screening of potential drugs is performed via heterologous expression of the target in cell lines. Potential hits, or druggable compounds, are then optimised and validated for their pharmacodynamics and toxicity and pass to the clinical phase. The potential integration of human genetic studies has helped greatly in the discovery of new potential targets, and the future use of human, patient-derived iPSCs for screening and the introduction of clearer biomarkers may lead to an optimised validation of the analgesic effect of new compounds to morph the current process into a more pathophysiology-driven one. **B** Ca^2+^ imaging holds the potential of acquiring a key role in the optimization of the drug discovery process (bolts in **A** show how Ca^2+^ imaging can bring “striking” changes at every stage of the current process). This approach can be applied, either *in vitro* or *in vivo*, to all stages of the drug discovery process, and at all levels of functional investigation to screen from single cells (B**i**) specific neuronal and non-neuronal sub-populations (B**ii**) to the whole animal (B**iii**) to have a complete picture of the effect of new analgesic compounds.

Considering this fluid, everchanging context, where the aim has shifted towards a better understanding of the pathophysiological mechanisms of pain in different diseases and disease models (human and surrogate), calcium imaging has the potential to acquire a central role in the struggle towards an improved drug design process in modern neuroscience (Fig. 3B). Modern preclinical target validation includes both *in vitro* and *in vivo* studies on multiple levels of investigation, from cell-to-cell populations and whole animals: calcium imaging has the means to bridge all these different levels (Seshadri et al., 2020). This technique, as illustrated above, has the advantage of increasing dramatically the throughput of the measures of neuronal activity both *in vitro* and *in vivo*, still providing excellent spatial resolution with minimal invasiveness and maintaining the integrity and physiology of the tissues in the case of *in vivo* experiments. This approach allows the researchers to study how gene mutations or target molecule silencing can have an impact on pain pathophysiology starting at the single cell level, using primary cultures from transgenic mice or patient-derived human induced pluripotential stem cells (Fig. 3Bi). These effects can then be verified *in vivo* on both peripheral and central structures in the nervous system.

Rodent pain models have been studied and validated over the years, and numerous models are still being generated and optimised to mimic human conditions better and better. Clinically relevant pain models like osteoarthritis, acute and chronic inflammation, cancer, neuropathies, and post-operative pain carry different efficacies, and require different behavioural readouts (Berge, 2014, Oertel and Lötsch, 2013, Sunil Kumar Reddy et al., 2012). The most used behavioural readouts are stimulus-evoked, with a mechanical (von Frey, Randall-Selitto, cotton swab), thermal (Hargreaves, Hot/Cold Plate, Place Preference, Acetone, tail or paw immersion) or chemical (Formalin, Capsaicin, Complete Freund’s Adjuvant, PGE_2_ as few examples) stimulus delivered to the hindpaw or tail of the rodents. These are fundamental in current pharmacological research and go invariably hand in hand with the choice of the drug to test (in terms of mechanism of action) and the pain model to be tested (Berge, 2014). Murine and human models utilise similar behavioural readouts and tools, like von Frey filaments, pinprick tweezers, iced and hot water, besides more complex instruments like Medoc TSA and the injection of irritants like capsaicin and PGE_2_ (Oertel and Lötsch, 2013, Okkerse et al., 2017, Serrano et al., 2017, van Amerongen et al., 2018). These kinds of stimuli and instruments can and have been used together with in *in vivo* calcium imaging experiments in the same fashion as their behavioural equivalents, alongside milder mechanical and thermal stimuli to explore somatosensation and pain simultaneously (Emery et al., 2016, Luiz et al., 2019, Wang et al., 2018a, Yarmolinsky et al., 2016). Furthermore, known, and novel analgesic drugs, and inflammatory or pain mediators can be delivered easily to the mouse during the imaging sessions, with either an intraplantar, intraperitoneal, subcutaneous, or intravenous injection to test the effect of these compounds on neuronal activity and helping define their functionality and site of action.

Finally, the use of transgenic mice, as stated above, has the great advantage to isolate the contribution of single neuronal subpopulations to different disease conditions (Fig. 3Bii). In this regard, of critical importance is the advent of cell specific neuropharmacology strategies, spanning from chemogenetic to optogenetic approaches (Iseppon and Arcangeletti, 2020, Mondoloni et al., 2019, Siuda et al., 2015). These approaches were initially designed to investigate the contribution of single ion channels and G-protein coupled receptors (GPCRs) to the physiology of cells and circuits, but more recent designs allow the investigation of the role of specific neuronal population in a wide variety of neurobiological context, pain being one of them. As an example, designer receptor exclusively activated by designer drug (DREADD) platforms are based upon GPCRs that are modified to be activated by a drug defined by the designer, usually clozapine-N-oxide (CNO) rather than their endogenous ligand. In principle, DREADDs can be produced using any GPCR, enabling for both the activation and the silencing of distinct neuronal subpopulations, selected via promoter-driven gene delivery ( Armbruster et al., 2007, Wood et al., 2020a). The level of precise spatio-temporal control that can be achieved with these neuropharmacological approaches is paramount in the progression of mechanistic studies towards a better replication and understanding of human pain physiology.

All these techniques can be integrated with *in vivo* calcium imaging: as an example, optogenetic activation of specific subpopulation has been combined with calcium imaging for the study of central nociception in the brain (Corder et al., 2019, Hu et al., 2019), and companies are developing miniaturized one or two-photon microscopes that allow the simultaneous optogenetic stimulation and recording of neural activity with calcium imaging (Inscopix - https://www.inscopix.com/). Moreover, DREADD chemogenetic modulation has been used in concert with behavioural assays, electrophysiology, and calcium imaging to study behavioural and functional changes in sensation and nociception (Nagai et al., 2020, Saloman et al., 2015), and can theoretically be used in concert with *in vivo* calcium imaging by injecting the drug during the acquisition phase of the experiment into mice already under anesthesia. These few examples show the potential for multiplexing and integration with diverse techniques that calcium imaging holds (Fig. 3Biii).

Besides the investigation of the mechanisms of sensation and pain, all these paradigms can be equally utilised to investigate the effect of new drugs and screen the effects the new compounds on the cellular and circuit function, with the advantage of a precise matching of neuronal activity with gene expression and phenotype specificity (Luiz et al., 2019, MacDonald et al., 2021a, Seshadri et al., 2020). Classic and novel analgesic compounds can be tested for their impact on neuronal function in peripheral DRGs, spinal cord neurons and in the brain, and this combination of *in vitro, ex vivo* and *in vivo* imaging with pharmacological treatment allows to visualise the efficacy as well as the effect of these drugs on cellular physiology, and in some cases to unravel novel analgesic mechanisms in a wide variety of surrogate models (MacDonald et al., 2021b, Wang et al., 2016).

## Limitations of calcium imaging and GCaMP probes

Calcium imaging and GCaMP probes are not free of disadvantages. While this technique enables observation of neural activity in real time with high spatial and temporal resolution and minimal invasiveness, it is indeed important to consider its limitations.

Calcium signals are paramount indicators of cellular activity, but they are an indirect measurement of electric activity. As stated before, electrophysiological approaches possess much higher temporal resolution and allow the recording of single and multiple APs and thus resolve several characteristics of neuronal activity such as their duration, number, and frequency. Such information is extremely difficult, if not impossible, to acquire from calcium signals, albeit many efforts in post-processing algorithms to estimate AP number and frequency from fast GCaMP data. Another issue of calcium imaging using GCaMPs is related to their temporal resolution: image acquisition is often not fast enough to resolve APs and there is potential for loss of information if an electrophysiological recording is not performed simultaneously. However, newer GCaMP probes have been produced in the very recent years to increase both probe sensitivity and kinetics and at least partially bridge this gap (Dana et al., 2019; “jGCaMP8 Fast Genetically Encoded Calcium Indicators,” n.d.).

Furthermore, GCaMP probes are heterologously expressed in neuronal cells, and their presence can alter the concentration of free calcium ions. This buffering effect can potentially lead to cellular changes that affect the vitality of the transgenic neurons (Tian et al., 2009). However, no cytotoxic effect has been shown to date in DRG neurons, especially in Pirt-GCaMP mice, one of the most used mouse lines (Anderson et al., 2018, Kim et al., 2014, Kim et al., 2016b). Indeed, GCaMP probes have the potential of modifying the activity patterns of neurons, and this is particularly important when imaging brain complex structures. A comprehensive study of the most used GCaMP-6 mouse lines found distinct clusters of aberrant activity patterns with high amplitude and short duration. Widefield calcium imaging showed these events are variable between different mouse lines and can affect the antero-lateral regions of the cortex, including the somatosensory and motor regions. Moreover, generalised seizures are observed in some of the mice from the more affected lines. Doxycycline treatment from birth, that prevents the expression of GCaMP-6, eliminates such events (Steinmetz et al., 2017).

Besides these biochemical and biological limitations of GCaMP probes there are still technical restrictions of calcium imaging altogether that must be considered thoroughly when designing an experiment. Anaesthesia, as previously stated, can be a limiting factor if the physiological impact on the tissue imaged is not taken into consideration. Furthermore, these are often terminal procedures, and although the anaesthesia can last for several hours, when studying the effects of analgesic drugs, one must consider the route of administration as well as the time needed to reach an effective concentration in the target tissue in relation to the vitality of the animal to be able to observe the changes in neuronal activity with respect to initial baseline. Finally, despite constant technical development, it remains heavily challenging to achieve stable imaging of highly myelinated tissues like the spinal cord.

## Conclusion

Calcium imaging is indeed a tool to explore pain pathophysiology in its totality, from basic cell studies to complex mechanistic investigation in whole animals. It allows us to dissect the effects of novel analgesics on single neuron and circuit physiology and evaluate the correct dosing linked to the desired action. Novel, exciting results have been produced utilising this approach, particularly *in vivo*, with new insights into the circuitry and mechanisms of peripheral and central nociception coming at a rapid pace. Considering the possibilities that come from the integration of all these different approaches with calcium imaging, this technique can be viewed as a powerful tool to build a comprehensive screening pipeline that integrates molecular biology and physiology, spanning all levels of investigation from single cell to the whole animal, and has the potential to be considered a pivotal step in the progress towards a more personalised and efficient drug discovery paradigm.

## Declaration of Competing Interest

The authors declare that they have no known competing financial interests or personal relationships that could have appeared to influence the work reported in this paper.
